# The role of DNA methylation in syndromic and non-syndromic congenital heart disease

**DOI:** 10.1186/s13148-021-01077-7

**Published:** 2021-04-26

**Authors:** Jiali Cao, Qichang Wu, Yanru Huang, Lingye Wang, Zhiying Su, Huiming Ye

**Affiliations:** 1 Department of Clinical Laboratory, Women and Children’s Hospital, School of Medicine, Xiamen University, Xiamen, 361003 People’s Republic of China; 2 Prenatal Diagnosis Centre, Women and Children’s Hospital, School of Medicine, Xiamen University, Xiamen, Fujian 361003 People’s Republic of China; 3 United Diagnostic and Research Center for Clinical Genetics, School of Medicine, Xiamen University, Xiamen, Fujian 361102 People’s Republic of China; 4 School of Public Health, Xiamen University, Xiamen, 361102 People’s Republic of China

**Keywords:** Congenital heart disease, DNA methylation, Genome methylation level, Differentially methylated regions, Maternal factors

## Abstract

Congenital heart disease (CHD) is a common structural birth defect worldwide, and defects typically occur in the walls and valves of the heart or enlarged blood vessels. Chromosomal abnormalities and genetic mutations only account for a small portion of the pathogenic mechanisms of CHD, and the etiology of most cases remains unknown. The role of epigenetics in various diseases, including CHD, has attracted increased attention. The contributions of DNA methylation, one of the most important epigenetic modifications, to CHD have not been illuminated. Increasing evidence suggests that aberrant DNA methylation is related to CHD. Here, we briefly introduce DNA methylation and CHD and then review the DNA methylation profiles during cardiac development and in CHD, abnormalities in maternal genome-wide DNA methylation patterns are also described. Whole genome methylation profile and important differentially methylated genes identified in recent years are summarized and clustered according to the sample type and methodologies. Finally, we discuss the novel technology for and prospects of CHD-related DNA methylation.

## Introduction

Congenital heart disease (CHD) is characterized by improper heart development manifesting as defects in the heart walls, valves or main blood vessels. The incidence of CHD in different studies varies from approximately 4/1000 to 50/1000 live births, and CHD is the most common type of congenital malformation [1, 2]. Unlike the lungs or kidneys, the heart is not composed of repeating functional units. The heart is an asymmetrical complex organ that is mainly composed of cardiomyocytes, fibroblasts, endothelial cells and perivascular cells [3]. The development of the heart is controlled by several overlapping morphogenetic systems that regulate complex cardiac transcriptional networks. Chromosomal abnormalities account for approximately 10–12% of all CHDs in live births [4, 5]; exome sequencing identified a set of de novo mutations in congenital heart disease, single mutations in specific morphogens or cardiac genes and related transcription factors have been shown to independently cause about 10% of severe CHDs [6, 7]; however, the etiology for the majority of cases remains unknown [2, 8]. A multifactorial pathogenesis for this disease with interplay between inherited and environmental causes has been proposed. To further improve the diagnosis, understanding and treatment of CHD, researchers must identify extragenomic factors that are essential for heart development.

Epigenetics refers to heritable processes that alter gene activity without changing the DNA sequence [9]. Three canonical mechanisms of epigenetic regulation are DNA methylation, histone modifications, and noncoding RNA activity [8]. The best known epigenetic process is DNA methylation, partially due to its mature detection methods [9]. As a switch to regulate gene expression, epigenetic regulation plays an essential role in differentiation and the maintenance of cell fate and is thus required for normal development and health, but epigenetic regulation is also responsible for some diseases [10]. Epigenetic changes that contribute to CHD have been summarized previously [2, 8, 11–13], and the correlations between dysregulated DNA methylation and CHD are described in detail here.

## DNA methylation

Although the genomes of major cell types in each individual are identical and fixed throughout the life cycle, epigenetic modifications are plastic and affect the spatiotemporal pattern of gene expression [14]. DNA methylation, which refers to the addition of methyl groups to DNA molecules, is one of the most important epigenetic mechanisms. This modification establishes and stabilizes cellular phenotypes by maintaining gene expression states [15, 16]. DNA methylation is required in many processes, such as embryogenesis, gametogenesis, X chromosome inactivation, imprinting, silencing of repetitive DNA elements, and regulation of gene expression and, therefore, cell function [17]. DNA methylation is an attractive topic in the study of complex diseases because it may be restored by environmental factors and dietary interventions [18–23]. DNA methylation present in gene promoters, gene bodies and repeated sequences has different effects. Abnormal methylation in promoter regions can inhibit transcriptional activity by preventing transcription factors from binding to target genes, which can cause gene silencing and diseases [24]. CpG islands have also been identified outside of promoter regions; in contrast, methylation of these islands sometimes increases transcription [25]. The development of DNA methylation detection technology has substantially promoted the understanding of the connection between DNA methylation and related diseases [26].

## Cardiac development and disease

The heart is the first organ to form during embryonic development. Heart development is a complex process that requires precise spatiotemporal control of multiple signals. Congenital heart diseases (CHDs) are malformations of the heart that appear during cardiac development [11]. Familial aggregation of CHD suggests that genetic factors play a role in CHD development. However, the majority of congenital heart malformations do not segregate in Mendelian ratios [27]. Other factors, such as the environment and epigenetics, also contribute to CHD development [2].

### Stages of heart development

Major insights into the cardiac development process were gained in studies of animal models, such as zebrafish, chickens and mice. The most primitive heart in the cephalochordate Amphioxus is just a contractile vessel, also called the tubular heart. Fish have two chambers: an atrium and a ventricle; amphibians and reptiles have three chambers. The hearts of humans, other mammals and birds consist of four separate chambers: left and right atria (upper half) and left and right ventricles (lower half) [28]. The development of the four-chamber heart has 4 main stages.

Myocardial cells are derived from the mesoderm. Myocardial markers are first detected between human embryonic days 12 and 15 (E12–E15), and two subsequent populations of cardiac mesoderm, termed the first heart field (FHF) and second heart field (SHF), migrate bilaterally away from the primitive streak and coalesce at the ventral midline of the embryo [29]. Is1/Gata4 and Foxh1/Nkx2.5 are at the top layer of the hierarchical order controlling SHF development [30]. The endocardial tubes fuse to form the primary linear heart tube at E20-E22, consisting of an inner layer of endocardium and an outer layer of myocardium [8]. The heart tube initially elongates linearly when new myocardial, endocardial and smooth muscle cells are added from the SHF to the two poles of the heart tube. During the third and fourth weeks, the linear heart tube undergoes rightward looping, and the poles of the heart tube continue to grow linearly, while the middle of the tube begins radial growth [31]. The heart begins to beat around this time. By E28, four heart regions—the atrium, atrioventricular canal (AVC), ventricle, and outflow tract (OFT)—become distinguishable. By approximately E50, the four heart chambers separate with the formation of four cardiac valves: the truncus arteriosus divides into the aorta and pulmonary artery, and the pulmonary veins and vena cava form and fuse with the left and right atria [32]. In addition to the complex morphological changes, the transformation of a heart tube to a mature heart requires extensive myocardial growth, including trabeculation, compaction, and thickening of the compact myocardium [33]. These stages are effectively summarized by Jarrell [8], Günthel [34], and Margaret Buckingham [29], and each stage has also been reviewed in detail [33, 35–40]. Minor errors at any stage may contribute to CHDs.

### Common types of congenital heart diseases (CHDs)

Dozens of distinct types of congenital heart defects are recognized, and the classification of CHDs can be found in previous reviews [1, 41]. These conditions can be divided into three main categories: heart valve defects, heart wall defects and blood vessel defects, depending on the site of the defect. Alternatively, they can be classified into cyanotic congenital heart disease and acyanotic congenital heart disease according to the levels of oxygen in the blood. In this section, five types of CHDs are briefly introduced.

The most common congenital heart defect is the bicuspid aortic valve (BAV). A bicuspid aortic valve has two instead of three cusps between the left ventricle and aorta. Approximately 1–2% of all people have a bicuspid aortic valve, as stated by Bo Yang, M.D., an assistant professor of cardiac surgery at the University of Michigan Frankel Cardiovascular Center, but most are not affected by valve problems until they are adults. Treatment may be required when the condition changes, such as valve problems or an enlarged aorta.

Ventricular septal defects (VSDs), another frequent CHD, exist in a hole in the wall separating the two lower chambers (ventricles) of the heart, which arises due to malalignment of the ventricular and OFT septa, and four types of VSDs can be distinguished anatomically, namely, muscular, central perimembranous, and outlet/inlet perimembranous VSDs [42]. Similar to VSDs, atrial septal defect (ASD) is a birth defect of the heart in which there is a hole in the wall (septum) that divides the atria of the heart. The hole lets blood from the left atrium mix with blood in the right atrium. Approximately 1 in every 1,859 babies born in the United States each year are born with ASD [43], and it comprises 30 to 40% of all congenital heart diseases found in adults [44].

TOF is an outflow tract (OFT or conotruncal) defect resulting from the incorrect alignment of the ascending aorta and pulmonary trunk with the left and right ventricles. The entire OFT is derived from the SHF, and impairment of SHF cell deployment results in shortening of the OFT, which then results in TOF [45]. TOF accounts for 10% of all congenital heart defects [46].

Double outlet right ventricle (DORV), in which both great arteries originate from the morphological right ventricle in a heart, is a rare form of CHD, accounting for 1–3% of all CHD cases [47–49]. In DORV, left ventricular drainage is usually achieved through different locations of ventricular septal defects and different relationships with the pulmonary artery and aortic outflow tract. Insufficiency of mitral valve and atrial septal defects (ASDs) can be found in DORV cases [50].

Abnormal development of any cardiac structure can lead to CHD. Improvements in diagnosis and surgical techniques have greatly improved the prospects of infants born with CHD.

### Causes of congenital heart disease

Families with autosomal dominant, recessive and X-linked CHDs have been reported [51–53]; however, genetic mechanisms have not been identified in the majority of cases. It is postulated that sporadic CHD, which accounts for approximately 80% of all cases, arises from an interaction of genetic and environmental factors [1]. Dysregulation of multiple genes and transcriptional pathways due to chromosome abnormalities, genetic mutations or dysregulated epigenetics contributes to congenital heart disease.

Chromosomal aberrations are frequently observed in CHD patients, especially those with syndromic CHD (CHD is one phenotype of the syndrome) [54]. The frequency of chromosomal abnormalities was approximately 12.9–23.1% [55]. Numerical and structural chromosomal abnormalities both contributed to CHDs. The most common numerical chromosomal abnormality in CHDs is trisomy of chromosome 21, which is also the cause of Down syndrome. Approximately 40–50% of patients with Down syndrome present with CHD [56]. Trisomy of chromosome 18 was the second most frequent trisomy. Trisomy X and monosomy X have also been observed. Structural changes in chromosomes, such as deletion of the short arm of chromosome 6 and duplication of the short arm of chromosome 17, were observed in septal defects [55]. CHD patients also show a higher incidence of many other syndromes existing chromosomal abnormalities or genetic mutations, including 22q11 deletion syndrome, Turner syndrome (complete or partial absence of the X chromosome), Williams syndrome (7q11.23 microdeletion), Heterotaxy syndrome (mutations in genes such as DNAH5, ZIC3, CFC1, NODAL, ACVR2B, DNAI1, and LEFTY2), Noonan syndrome (mutations in genes associated with the RAS-MAPK signaling pathway, such as PTPN11, SOS1, RAF1, KRAS, NRAS, BRAF, SHOC2, and CBL), Marfan syndrome (mutations in FBN1 gene), Alagille syndrome (mutation in Notch signaling pathway genes JAG1 and NOTCH2), CHARGE syndrome (mutation in CHD7 gene) [56].

Genetic studies have identified a set of transcription factors that maintain the identity of differentiated myocytes. For example, COUP-TFII is an orphaned nuclear hormone receptor expressed in atrial myocytes but not in ventricular myocytes [57]. This molecule is involved in restricting ventricular identity and promoting atrial identity by controlling a large range of genes important for atrial and ventricular identity [58]. Conversely, the transcription factors NKX2.5, HEY2 and IRX4 are important for maintaining ventricular chamber identity. The critical signaling pathways and transcription factor networks that regulate cardiomyocyte lineage specification are summarized by Sharon L. Paige [59] and Benoit G. Bruneau [60], and the genetic basis of congenital cardiovascular malformations is also highlighted by Seema R. Lalani [5] and Troels Askhøj Andersen [61]. Dysregulated expression of these genes hampered the proper formation of cardiac structure. Exome sequencing conducted by Alejandro Sifrim et al. identified distinct genetic architectures for syndromic and nonsyndromic CHD. In syndromic CHD, a significant enrichment of de novo protein-truncating variants, but not inherited protein-truncating variants, in known CHD-associated genes was observed. Conversely, significant enrichment of protein-truncating variants inherited from unaffected parents in nonsyndromic CHD [62].

More than 55 human genes have been shown to be involved in CHDs [61]. For example, the NOTCH pathway is a paradigmatic endocardial-derived signal that regulates cell fate specification and tissue patterning to define chamber versus valve domains in the early vertebrate heart [63]. Defective endocardial NOTCH signaling affects cardiac valve and chamber development, leading to diseases such as bicuspid aortic valve (BAV) and left ventricular noncompaction (LVNC) cardiomyopathy [63–65]. Loss-of-function mutations in TBX20 can cause dilated cardiomyopathy, atrial septal defects, or mitral valve disease, while gain-of-function mutations in TBX20 have been reported in patients with tetralogy of Fallot [66–70]. According to the function of genes, these pathogenic genes can be divided into 5 categories: genes associated with laterality defects (e.g., ACVR2B, CFC1, CITED2, GDF1, LEFTY2, NODAL and ZIC3), genes encoding components of signaling pathways (typically the NOTCH pathway and the MAPK pathway), genes encoding cardiac transcription factors (e.g., BMP2, GATA4, NKX2-5, TBX20, TBX2 AND TBX5), genes encoding components of the cardiac sarcomere (e.g., ACTC1, MYH6, MYH7 and MYH11) and genes encoding chromatin modifiers (e.g., CHD7, KMT2D, EP300, CREBBP and EHMT1) [61].

Despite significant progress in understanding heart development and diagnosing heart disease, the striking complexity of the heart continues to limit our ability to identify the molecular mechanisms of CHD. In addition to genetic factors, epigenetics and maternal factors affect the development of the heart, which will be discussed in the following sections. For many cases, the pathogenesis remains to be explored.

## Maternal DNA methylation patterns and congenital heart defects

It is known that maternal factors have maximum influence on fetal growth. Maternal age, gestational diabetes, obesity, dietary deficiencies, cigarette smoking, alcohol intake, exposure to teratogenic medications, and viral infections have been reported as maternal-environmental risk factors for the development of CHD [71]. How these environmental factors contribute to CHD is a difficult question that remains to be solved. Changes in DNA methylation patterns may be one mechanism for these risky environmental factors.

Prognostic and predictive significance of long interspersed nucleotide element-1 (LINE-1) methylation has been proposed in several diseases, such as advanced-stage colorectal cancer and high-grade cervical intraepithelial neoplasia [72, 73]. Maternal LINE-1 DNA hypomethylation has been found to be associated with an increased occurrence of nonsyndromic CHDs. Measurement of maternal DNA methylation in a case–control study of CHDs assessed the relationship between LINE-1 DNA methylation and the risk of CHDs, and the findings indicated that maternal LINE-1 hypomethylation is associated with an increased risk of CHD-affected pregnancies [74]. Significantly lower levels of LINE-1 methylation in the mothers of children with Down syndrome compared to mothers of healthy children were also observed [75]. However, the association between maternal LINE-1 methylation and CHD in children with Down syndrome was not found in a study consisting of 44 mothers of children with Down syndrome and CHD and 46 mothers of children with DS without CHD (P = 0.997) [76] (Table 1). Shimul Chowdhury et al. conducted another study to determine if alterations in gene-specific methylation were associated with CHDs. Genome-wide maternal DNA methylation analysis of these samples identified 425 CpG sites encompassing 415 genes that were differentially methylated between cases and controls (P < 0.005). Case mothers showed hypermethylation in most differentially methylated loci (379 sites, 89.2%) compared to hypomethylated loci (46 sites, 10.8%) [77]. Multiple genes involved in the mitogen-activated protein kinase (MAPK) pathway and EGFP, GATA4, and Wnt5a, which are implicated in heart development, were differentially methylated in this study [74, 78–80].

**Table 1:** Differently Maternal DNA methylation patterns and congenital heart defects

Study (reference)	Study population Sample type	Cohort	Sample size	Methylation measures	DNA methylation change
[74]	Mothers, Blood	non-syndromic CHD	Case: 180, Control:187	Methylation of LINE-1 assessed with Methylight was used as a surrogate marker of global DNA methylation status	LINE-1 DNA methylation was significantly lower in cases compared with controls (* P* = 0.049)
[76]	Mothers, Blood	CHD with Down syndrome	Case: 44, Control: 46	LINE-1 DNA methylation was analyzed by quantification of LINE-1 methylation using the MethyLight method	LINE-1 methylation was not significantly different between DS-CHD + and DS-CHD − mothers (*P* = 0.997)
[77]	Mothers, Blood	non-syndromic CHD	Case: 180, Control:187	Maternal gene specific methylation in over 27,000 CpG sites was assessed using the Illumina Infinium Human Methylation27 BeadChip	425 CpG sites encompassing 415 genes were differentially methylated between cases and controls (P < 0.005). The majority of differentially methylated CpG sites were hypermethylated in cases

The roles of maternal nutritional epigenetics in congenital heart disease have been well summarized by Radha O Joshi et al. The influence of diet quality on the incidence of heart defects were observed in mouse, rat and population-based study. The maternal diet during the gestation period is considered to alter the DNA methylation status globally or at specific loci by affecting direct provision of methyl donors or by providing cofactors of DNMTs and enzymes that regulate the one-carbon cycle [81]. Some related studies suggest the linkage of maternal risk factors and abnormal DNA methylation, and further CHD. Folate is a dietary B vitamin that provides methyl groups for DNA methylation and other methylation reactions in cells. Genomic DNA methylation directly correlates with folate levels. Folate deficiency is known to cause hyperhomocysteinemia, which is a risk factor for the development of CHD. A population-based case–control study of 572 women with CHD-affected pregnancies and 363 control women indicated that functional polymorphisms in folate-related genes increase the risk of having a fetus with CHD when maternal lifestyle factors that alter folate metabolism are present. Methylene tetrahydrofolate reductase (MTHFR) is an enzyme important for folate metabolism [82]. Retrospective studies have shown that the MTHFR C677T mutation is associated with up to 50% of certain types of CHD [83]. The effect of maternal obesity on the developing heart appears to be greatest among women who carry the MTHFR C677T or BHMT G742A polymorphism. Maternal smoking and/or alcohol use in combination with the TCII C776G polymorphism may increase the risk of CHDs [84]. Another study also found that the MTHFR genotype/diet combination and BMI were significantly associated with LINE-1 methylation in mothers of children with Down syndrome and CHD [76]. These findings are consistent with the role of DNA methylation in the development of CHD.

Early diagnosis and noninvasive diagnosis of fetal diseases using maternal samples are aspirational; although the complicated physical conditions of pregnant women make these processes highly difficult, they are still a valuable research direction. Alterations in maternal DNA methylation may exert effects through direct interactions with the fetus or indirectly affect the fetus through changes in the intrauterine environment. Changes in the maternal environment can result in direct changes in gene expression in the developing fetus [77, 85, 86]. Based on knowledge of genetic susceptibility, lifestyle factors and their interactions, risk stratification of women of childbearing age may play an important role in reducing adverse pregnancy outcomes, including heart defects and other structural malformations. Alterations in maternal DNA methylation can be achieved via nutritional intervention, making DNA methylation an attractive potential therapeutic target [74].

## DNA methylation orchestrates cardiomyocyte development, maturation and disease

The development of the heart is a complicated process. DNA methylation plays a key role in the fine-tuned spatiotemporal expression of cardiac genes during development. Cardiac DNA methylation differs in different embryonic stages, neonates and adults, healthy persons and patients.

### Dynamic DNA methylation profiling in the development of heart

During mammalian differentiation and development, DNA methylation undergoes remodeling to produce a cell type-specific methylation pattern found in adult somatic cells. DNA is demethylated within each developing germ cell and then specifically remethylated during gametogenesis [87–89]. Several reports have shown that the paternal genome undergoes genome-wide DNA demethylation before replicating its DNA during the development of mouse zygotes [90–92]. It was also observed in human zygotes that paternal chromatin underwent active demethylation soon after fertilization [93]. Single-cell DNA methylation sequencing of human preimplantation embryos revealed that thousands of genomic loci exhibit de novo DNA methylation [94]. These reports indicate that genome-wide DNA methylation reprogramming during embryo development is a dynamic balance between global demethylation and focused methylation. The epiblast genome is remethylated rapidly after implantation [95, 96]. Whole-genome surveys conducted in mice and humans have shown that there are extensive and focal differences in DNA methylation patterns among various adult tissues, which indicate that discrepancies in de novo DNA methylation and demethylation occur during tissue differentiation [97–99].

DNA methylation is dynamic during cardiomyocyte development. Several studies on dynamic DNA methylation profiling in the development of heart was summarized in Table 2. A genome-wide DNA methylation profiling of mouse embryos showed that global methylation of 1.64 million ACGT sites in developing hearts remained stable between embryonic day (E) 11.5 and E14.5, and a small fraction (2901) of them exhibited differential methylation (1946 showed increased methylation and 955 showed decreased methylation in late-stage hearts); these sites were enriched in genes involved in heart development, including ERBB4, GATA6, FOXP1, FGF2, FGF9, HAS2, INVS, MEF2C, ROBO2 and WNT2. Of the 350 genes analyzed by quantitative real-time PCR, 79 genes had correlative changes between methylation and expression [100]. Cardiomyocytes contained short regions of low DNA methylation, which showed characteristics of cis-regulatory elements compared with those of ES cells. Five percent of CpGs were differentially methylated between cardiomyocytes of healthy adult mice and undifferentiated ES cells. Among them, 90% of these CpGs were hypomethylated and 10% were hypermethylated in cardiomyocytes versus ES cells, which indicated that differential CpG methylation showed cell type specification during embryonic development. Demethylated regions of adult cardiomyocytes were significantly enriched for upstream transcription factor binding motifs (TF motifs) of known cardiac transcription factors, including MEF2C and GATA1-4 [101]. A comparation of heart from embryonic, newborn and adult mouse found that DNA methylation levels of the CpG dinucleotides region in ssTnI gene promoter were increased with the development of mouse heart, corresponding to a decreased expression of ssTnI gene. These results indicate that DNA methylation is involved in regulation of myofibril gene expression during heart development in mice[102].

**Table 2:** Dynamic DNA methylation profiling in the development of heart

Study (reference)	Study population Sample type	Cohort	Sample	Methylation measures	DNA methylation change
[100]	Heart tissue (mouse)	Healthy	E11.5, E14.5 (*n* = 3 per group)	Genome‐wide DNA methylation profiling using MSFE/MPS	Global methylation in developing hearts remained stable between E11.5 and E14.5, a small fraction AGCT sites exhibited differential methylation, which were enriched in genes involved in heart development
[102]	Heart tissue (mouse)	Healthy	E14.5, E17.5, NB, P7, P14, adult	The DNA methylation levels of CpG dinucleotides region in ssTnI gene promoter were detected using MSP and BSP	DNA methylation levels of the CpG dinucleotides region in ssTnI gene promoter were increased with the development of mouse heart
[103]	Heart tissue (mouse)	Healthy	P1, P7, P14, P28, P84 (*n* = 3 per group)	For analysis of global DNA methylation, 5‐methylcytosine levels were measured using the MethylFlash Methylated DNA Quantification Kit	Majority of DMRs (~ 80%) were hypermethylated between P1 and P14, and hypermethylated regions were associated with transcriptional shut down of important developmental signaling pathway
[101]	Heart tissue (mouse)	Chronic pressure overload in adult mice	newborn, adult healthy and failing hearts (*n* = 3–6)	DNA methylome were analyzed by whole-genome bisulfite sequencing	Identified large genomic regions that are differentially methylated during cardiomyocyte development and maturation

DNA methylation is also highly dynamic during postnatal maturation. Genome-wide sequencing of methylated DNA conducted by Choon Boon Sim et al. identified dynamic changes in the cardiac methylome during postnatal development (2545 DMRs from P1 to P14 in the mouse). Majority of DMRs (~ 80%) were hypermethylated between P1 and P14, and these hypermethylated regions were associated with transcriptional shut down of important developmental signaling pathways, including Hedgehog, bone morphogenetic protein, TGF-β, fibroblast growth factor, and Wnt/β-catenin signaling [103]. A substantial number of CpGs were differentially methylated in newborn and adult cardiomyocytes [101]. Gene body demethylation starting at the 5′ end and extending to the 3′ end was observed during postnatal maturation of cardiomyocytes; for example, the 5′ end of the ATP2A2 gene and upstream TF motifs were already demethylated at birth, while the 3′ region lost its CpG methylation by adulthood, which was correlated with increased expression of ATP2A2. However, the demethylated cardiomyocyte genes could be repressed by trimethylated H3K27 (H3K27me3), which could inhibit transcription and de novo CpG methylation. For example, ISL1 (insulin gene enhancer protein ISL-1, a marker for cardiac progenitors of the secondary heart field) and PITX2 (paired-like homeodomain 2, responsible for the asymmetrical development of the heart and expressed in tubular heart) are stably repressed by H3K27me3. De novo CpG methylation of skeletal troponin I isoform (Tnni1) by DNMT3A/B was accompanied by the demethylation of TNNI3 during heart maturation and mediated the transition from fetal to adult sarcomeric gene expression [101]. From the fetal stage to adulthood, the decrease in DNA methylation is associated with increased expression of genes responsible for myofibril or sarcomere structures, while the increase in DNA methylation is associated with decreased expression of primarily developmental genes, including MYC, SOX11, TBX5 and TNNI1 [104].

The importance of cell-type-specific epigenomic analyses was highlighted [105]. Fetal and infantile hearts predominantly consist of cardiac myocytes (CMs). However, CMs loose cell cycle activity, endothelial and mesenchymal cell proliferation augments after birth [106]. Ralf Gilsbach et al. purified CM nuclei from prenatal and postnatal human hearts by labeling of cardiac nuclei with anti-PLN antibodies. Analysis of fetal, infantile, and adult non-failing data sets revealed dynamic genic mCpG during prenatal and postnatal development affecting 10% of all genes harboring genic regions with unmethylated CpGs (gUMR). Alterations within these genes were also present on the layer of histone modifications and gene expression and affect especially genes involved in the maturation of CMs. From fetal life until adulthood, 529 genes exhibited differential gUMR mCpG (DM-gUMRs). Loss of mCpG in DM-gUMRs was associated with increased expression of genes essential for myofibril and sarcomere structures as well as regulation of contraction, while a developmental increase of mCpG in DM-gUMRs was linked to decreased gene expression and affected primarily developmental genes. From fetal to infant stage, 29 gUMRs were hypomethylated and 12 gUMRs were hypermethylated. Postnatally, 188 gUMRs were further demethylated and 34 gUMRs were hypermethylated. In all, genes that were differentially methylated prenatally showed continuing methylation changes postnatally [104, 107].

### Abnormal DNA methylation profiling in CHD

Methylation levels of failing murine hearts caused by chronic left ventricular pressure overload partially resembled the newborn CpG methylation pattern. Failing murine heart-associated differentially methylated regions (DMRs) are mostly intergenic, and their overlap with postnatal DMRs is adjacent to genes involved in cardiac muscle cell development, cardiac morphogenesis and energy metabolism [101]. For humans, decreased expression of DNMT1 and DNMT3B was observed in TOF patients, which may be associated with global DNA hypomethylation and play an important role in the pathogenesis of TOF [108]. In another study, 14,403 hypermethylated DMRs and 1450 hypomethylated DMRs were detected in TOF cases compared to controls (normal hearts), and for VSD cases, hypermethylated DMRs and hypomethylated DMRs were 7152 and 935, respectively. More hypermethylated than hypomethylated tiling regions were observed in patients with TOF (hyper/hypomethylated: 88.3/11.7%) or VSD (hyper/hypomethylated: 76.2/23.8%) compared to normal heart controls [109]. In the same TOF cases and controls (right ventricle of normal hearts), 978 autosomal protein-coding genes were significantly differentially expressed. Of these genes, 38% were upregulated and 62% were downregulated in TOF. Hypermethylated promoters were significantly associated with downregulated (*P* = 0.01) gene expression, while hypomethylated promoters were significantly associated with upregulated (*P* = 7.2 × 10^−5^) gene expression. Investigation of DNA methylation in gene bodies (without first exons) also revealed a significant overlap between upregulated genes and hypomethylated exons (*P* = 0.02) [109]. For both TOF and VSD, significant hypermethylation in the promoter of the SCO2 gene was detected, and quantitative real-time PCR showed a significant reduction in SCO2 expression in myocardial biopsies of both patient groups. It was hypothesized that the downregulation of SCO2, which is caused by hypermethylation, drives the metabolic state toward glycolysis, delaying terminal differentiation and promoting cardiomyopathy and heart failure. Significantly down-regulated gene with a hypermethylated promoter was also observed in ECE2, a gene thought to be important in patterning cardiac neural crest cell derivatives [109]. Upregulated hypomethylation in the promoter of TDGF1 was also observed in TOF patients [109]. TDGF1, also named CRIPTO, is the prototypic member of the epidermal growth factor-like cripto-FRL-1-cryptic (EGF-CFC) family and can induce cell proliferation, reduce apoptosis, and increase cell migration and is also an important signaling factor during cardiogenesis [110]. The TDGF1 gene plays an essential role in not only specification but also maintenance and differentiation of early cardiac progenitors [111].

Clara Serra-Juhé et al. explored the global methylation profile of fetal heart DNA in comparison to blood DNA from control subjects and detected significant enrichment of differential methylation at genes related to muscle contraction and cardiomyopathies in the developing heart DNA. They also searched for abnormal methylation profiles on developing heart-tissue DNA of syndromic and non-syndromic congenital heart defects. Hypermethylation of several intragenic sites at the MSX1, a gene involved in outflow tract morphogenesis by protecting secondary heart field precursors against apoptosis and by inhibiting excessive proliferation of cardiac neural crest, endothelial and myocardial cells in the conotruncal cushions, was found in a fetus with iCHD. Hypermethylation of the GATA4 gene was present in fetuses with Down syndrome with or without congenital heart defects, as well as in fetuses with iCHD [112]. The inactivation of GATA4 was hypothesized as a cause of CHD induced by abnormal hypomethylation and low expression of BRG1. Transcriptional activator BRG1, also known as ATP-dependent chromatin remodeler SMARCA4, is necessary for cardiac development and heart function [113]. BRG1 deficiency resulted in heart defects in mice, and BRG1 mutations are found in CHD patients [114, 115]. The CpG shore of BRG1 in the second intron was reported to be hypomethylated in the myocardium of CHD patients, and BRG1 presented a low expression level [116, 117]. The study of Dobosz A. et al. compared the gene promoter methylation profiles between two groups of Down syndrome patients, with and without heart defects of endocardial cushion-type and detected significant hypermethylation of the NRG1 gene promoter region in children with heart defects [118].

Decreased LINE-1 methylation levels were found in the cardiac tissue of TOF patients in an analysis of 32 TOF patients and 15 controls in the study of Wei Sheng et al. [119]. It has been hypothesized that LINE-1 hypomethylation may promote carcinogenesis by facilitating chromosomal instability and controlling gene expression [120]. These processes may also be associated with an increased risk of TOF. ROC curve analysis confirmed the accuracy of predicting the presence of TOF by the LINE-1 methylation level, which provides initial evidence of the association between the LINE-1 methylation level and TOF in pediatric patients [119]. Wei Sheng et al. also found that TOF patients had significantly higher methylation levels in the promoter CpG islands of NKX2-5 and HAND1 than controls and lower methylation levels in the promoter CpG island of TBX20 [119]. Quantitative methylation analysis of the NKX2-5, GATA4 and HAND1 genes in the right ventricular myocardial tissues of 10 TOF patients and 6 age-matched control subjects found significant changes in the methylation levels in the NKX2-5 gene body and the HAND1 gene promoter region. The aberrant methylation status of NKX2-5 and HAND1 was negatively correlated with their corresponding transcription levels [121]. NKX2-5 and HAND1 both encode transcription factors that regulate specific phases of heart development [122]. The DNA methylation change in these genes leads to variation in transcriptional regulation and may play important roles in the pathogenesis of TOF [121]. In another study of Wei Sheng et al., methylation status analysis on the promoter regions of 71 CHD candidate genes of 10 TOF cases and 6 controls was performed and significant differences in methylation status in 26 genes were found. Seven genes were validated in 41 TOF cases because of their nominally significant differences in methylation levels and their function in heart development. The methylation values of EGFR, EVC2, TBX5 and CFC1B were significantly correlated with their mRNA levels, indicating that aberrant methylation changes of specific genes may contributes to the development of TOF [123].

Cardiac tissue is inaccessible in living fetuses and infants, so analysis using surrogate tissue such as blood could dramatically enhance our ability to detect and evaluate CHD [124, 125]. Changes in the methylation level of CHD patients in blood and placenta are also summarized (Table 3). Compared to that in newborn healthy infants, a total of 52 genes, corresponding to 59 CpG sites, with significantly altered DNA methylation were identified in the blood of newborn AVS patients [124]. Among the 52 genes identified, DUSP27 (dual specificity phosphatase 27), RUNX1 (runt-related transcription factor 1) and TXNRD2 (thioredoxin reductase 2) play an important role during muscle and heart development [126–128], APOA5 and PCSK9 are associated with cardiovascular disease[129–131], while many have not been reported to be associated with AVS in previous studies. These results also suggest that the selected genes may be involved in the development of AVS by DNA methylation [124]. A genome-wide methylation assay in newborn blood from 24 nonsyndromic TOF cases and 24 unaffected matched controls identified 64 significantly differentially methylated CpG sites, of which 25 CpG sites had high predictive accuracy for TOF. Multiple differentially methylated genes, such as ABCB1, CREM, LHX9, PPP2R5C, RUNX, SELL, SCN3A and TLR1, in the blood DNA of newborns with TOF are known or plausibly linked to heart development and postnatal heart disease [125]. Genome-wide DNA methylation analysis of newborn blood DNA from 24 nonsyndromic coarctation of aorta (CoA) cases and 16 controls identified significant methylation changes in 65 different CpG sites located in 75 genes. Genes important for heart development, for example, TGFB1, SMAD1 and glucocorticoid signaling-related genes, were found to have significant methylation changes in CoA [132]. A comparation of DMRs between DS with and without CHD by Benjamin I Laufer et al. distinguished 1588 nominally significant DMRs (35% hypermethylated, 65% hypomethylated). GO enrichment analyses revealed significant enrichments for terms related to the heart, including atrial cardiac muscle tissue development and actin-based cell projection. Notably, there was an 880 bp hypomethylated DMR that mapped to RUNX1 in CHD compared to non-CHD DS cases [133].

**Table 3:** Abnormal DNA methylation profiling in CHD

Study (reference)	Study population Sample type	Cohort	Sample size	Methylation measures	DNA methylation change	
[109]	Myocardial tissues (right ventricle/right atrium)	TOF: 8, VSD: 8	Case: 16, Control: 7	DNA methylation profiles were generated using MBD-seq	A significant overlap for hypermethylated promoters and down-regulated genes was found, and vice versa. A hypermethylated CpG island in the promoter of SCO2 was identified	
[112]	Fetal heart tissue	6 DS without CHD, 6 DS with CHD, 6 isolated CHD	Case: 18, Control: 4	Global methylation profile was studied by using the Illumina Infinium Human Methylation27 array Platform	Hypermethylation of several intragenic sites at the MSX1 gene was found in iCHD Hypermethylation of the GATA4 gene was present in Down syndrome with or without congenital heart defects, as well as iCHD	
[119]	Children (1 to 48 months), Myocardial tissues (right ventricle)	TOF	Case: 32, Control: 15	Sequenom MassARRAY platform was performed to examine the methylation levels of LINE-1, NKX2-5, HAND1 and TBX20	Lower LINE-1 methylation levels are associated with increased risk of TOF. Higher methylation levels of NKX2-5 and HAND1 and lower methylation levels of TBX20 were observed in TOF patients than in controls	
[124]	New born (24 to 79 h), Blood	AVS	Case: 24, Control: 24	A genome-wide DNA methylation analysis using an Illumina Infinium 450 k human methylation assay was conducted	The study identified significantly-altered CpG methylation at 59 sites in 52 genes in AVS subjects as compared to controls, including DUSP27, RUNX1, TXNRD2, APOA5 and PCSK9	
[125]	New born (24 to 79 h), Blood	TOF	Case: 24, Control: 24	Genome-wide methylation assay used Illumina Infinium HumanMethylation450 BeadChips	64 differentially methylated CpG sites in TOF cases were identified. Multiple differentially methylated genes are known or plausibly linked to heart development and postnatal heart disease	
[133]	New born (24 to 72 h), Blood	DS patients with or without CHD	DS patients with CHD: 11, without CHD: 10	Low-pass whole genome bisulfite sequencing (WGBS) of 86 NDBS DNA was performed to examine DNA methylation profiles	A comparation of DMRs between DS with and without CHD distinguished 1588 nominally significant DMRs (35% hypermethylated, 65% hypomethylated). There was hypomethylated DMR mapped to RUNX1 in CHD	
[49]	Blood	DORV	Monozygotic twins, Case: 1, Control: 1	The DNA methylation pattern were analyzed using reduced representation bisulfite sequencing	Many DMR-related genes are enriched in pathways that contribute to cardiac development, such as ZIC3 and NR2F2	
[139]	Blood	TOF	Monozygotic twins, Case: 2, Control: 2	Whole genome sequencing (WGS) and whole genome bisulfite sequencing (WGBS) were used to examine the genetic and epigenetic differences	Difference between the two monozygotic discordant twin pairs was observed in epigenetic alterations rather than at the genetic or structural genomic level, such as DMCs in the promoter region of the cardiac transcription factors TBX20, GATA4 and NKX2-5	
[12]	Blood	HLHS: 8, VSD: 8, ASD: 12, CoA: 14, PS: 14, ToF: 14	Case: 60, Control: 32	Genome-wide DNA methylation analysis was performed on DNA from newborn blood spots	Profound differences in cytosine methylation were observed in hundreds of genes in newborns with different types of CHD	
[156]	Fetal skin fibroblasts	DS with CHD (VSD, ASD)	DS MZ twins discordant for CHD, Case: 4, Control: 4	Reduced Representation Bisulphite Sequencing (RRBS) were applied to profile DNA methylation changes	197 DMRs in monozygotic twins discordant for VSD and 88 DMRs in monozygotic twins discordant for AVSD were identified, including 13 common DMRs between two sets	
[141]	Placenta	VSD	Case: 8, Control: 10	Genome-wide DNA methylation assay was performed using the Illumina HumanMethylation450 BeadChip assay	Most differentially methylated genes, have been shown to be associated with heart development or disease	
[142]	Placenta	TOF	Case: 8, Control: 10	The Illumina Infinium HumanMethylation450 BeadChip assay was used	ARHGAP22, CDK5, TRIM27 and IER3 had outstanding predictive accuracy for the prediction of TOF. ID4, HES7 and PPP3CA, which may be associated with heart disease, also showed differential methylation in this research	
[116]	Myocardium	TDF, VSD, DCRV	Case: 24, Control: 11	The methylation of a BRG1 promoter was analyzed using pyrosequencing and the MassARRAY platform	BRG1	hypomethylation
[149]	Myocardial tissues	CHD	Case: 31, Control: 2	BSP and MSP were used to detect the methylation in CITED2 promoter region	CITED2	hypermethylation
[143]	Blood	VSD, ASD, TOF, PDA, CoA, other CHDs	Case: 27, Control: 28	Eighteen imprinted genes methylation were measured by matrix-assisted laser desorption/ionization time-of-flight mass spectrometry	GRB10, MEST	hypermethylation
					PEG10, NAP1L5, INPP5F, PLAGL1, NESP, MEG3	hypomethylation
[144]	Blood	CHD with EM	Case: 24, Control: 20	The methylation levels of 3 candidate genes were analysed by the MassARRAY platform	SNRPN, ZAC1	hypermethylation
					INPP5F	hypomethylation
[118]	Blood	AVSD, VSD	Case: 7, Control: 9	Methylation analysis of the promoter regions was performed by means of the Human Promoter 1.0 Arrays (Affymetrix)	NRG1	hypermethylation

Disease-discordant monozygotic twin pairs share nearly identical genetic variants, so they are outstanding subjects to study epigenetic mechanisms driving pathologies [134–136]. The DNA methylation pattern in the pathogenesis of DORV, analyzed through DNA methylation profiling of whole-blood DNA derived from a monozygotic twin pair discordant for DORV at the single nucleotide level, provided evidence for the presence of epigenetic differences between the twin pair. Many DMR-related genes are enriched in pathways that contribute to cardiac development, such as ZIC3 and NR2F2. ZIC3 and NR2F2, which are both associated with CHD in the OMIM database, were hypermethylated upstream of the transcription start sites (TSS) in the hearts of patients with DORV compared to normal hearts [49]. ZIC3 is a transcription factor that functions in the early stages of left–right body axis formation and heart development [137]. NR2F2 has been crucially implicated in angiogenesis and heart development in humans and mouse models, and abnormal expression or depletion of NR2F2 has been reported to lead to AVSD (atrioventricular septal defect) and VSD [138]. Aberrant methylation at promoter regions of ZIC3 and NR2F2 and their dysregulated transcription levels may contribute to DORV [49]. In this study, DMRs were also found upstream of CITED1, GATA2, SOX3 and some important epigenetic genes, including MTA2, NSD1, MECP2 and SUV39H1 [49]. In Marcel Grunert’s study, genome-wide high-throughput sequencing was used to examine the genetic, structural genomic and epigenetic differences of two identical twin pairs discordant for TOF, and the results showed that the difference between the two monozygotic discordant twin pairs was observed in epigenetic alterations rather than at the genetic or structural genomic level, such as DMCs in the promoter region of the cardiac transcription factors TBX20, GATA4 and NKX2-5 [139]. The study of M. Reza Sailani et al. identified epigenetic changes in genes with potential involvement in heart development in Down syndrome monozygotic twins discordant for CHD. The two twins set in this study were discordant for VSD and AVSD. Although genomic variation on chromosome 21 has a high correlation with the risk of CHD, the genome is similar in the case of monozygotic twins. 197 DMRs in monozygotic twins discordant for VSD and 88 DMRs in monozygotic twins discordant for AVSD were identified, including 13 common DMRs between two sets [140].

A genome-wide DNA methylation assay of the placentas from 8 patients with isolated VSD and 10 unaffected controls identified significant epigenetic dysregulation in the placental DNA of VSD cases and identified 80 highly accurate potential CpGs in 80 genes for the detection of VSD [141]. The biological processes and functions of most differentially methylated genes, including ACTC1, HEYL, HEY2, ISL1 and SRF, have been shown to be associated with heart development or disease [141]. A total of 165 significantly differentially methylated CpG loci in 165 separate genes in TOF cases compared to controls were also found by analyzing placental tissue obtained at birth from 8 cases with nonchromosomal, nonsyndromic TOF and 10 unaffected newborns. ARHGAP22, CDK5, TRIM27 and IER3 had outstanding predictive accuracy for the prediction of TOF (AUC ≥ 0.95). ID4, HES7 and PPP3CA, which may be associated with heart disease, also showed differential methylation in this research [142]. Placental tissue obtained at birth may also be used for the prediction of newborn CHD [141, 142]. Distinct DNA methylation patterns observed in patients with CHD are being studied as potential biomarkers for CHD detection, and noninvasive surrogates may be valuable in the diagnosis of CHD.

Distinct DNA methylation patterns have been observed not only in 60 CHD patients compared to 32 healthy persons but also in various CHD types, suggesting that these signatures could be used to discriminate between specific CHDs. A panel of CpG methylation biomarkers for the prediction of individual types of CHD was identified by Ray O. Bahado-Singh et al. [12]. In the study of M. Reza Sailani et al., DMRs in EP300, E2F6, SMC3 and CEBPB binding sites were highlighted in Down's syndrome twins discordant for AVSD, while for Down's syndrome twins discordant for VSD highlighted POLR2A (polymerase (RNA) II polypeptide A), CTCF and EP300 [140]. However, the assay was based on a relatively small number of subjects. Additional genome-wide methylation scans performed in larger cohorts are required to confirm the potential of using variation in CpG methylation levels as biomarkers for CHD.

Many of the genes reported to be involved in the development of the heart exhibited differential methylation in CHD patients. Epigenetic regulation of imprinted genes is involved in many embryonic developmental processes, including cardiac development. Shaoyan Chang et al. investigated the alterations of eighteen imprinted genes germline differential methylation regions (gDMRs) methylation in patients with CHD and found altered gDMR methylation level of 8 imprinted genes, including 2 imprinted genes with hypermethylation of GRB10 and MEST and 6 genes with hypomethylation of PEG10, NAP1L5, INPP5F, PLAGL1, NESP and MEG3. Risk analysis showed that 6 of these genes (except MEST and NAP1L5) within a specific methylation level range were the risk factors for CHD [143]. Another study on aberrations imprinting genes involved in the pathogenesis of CHD with extracardiac malformations also found the hypermethylation of INPP5F. In addition, risk analysis showed that abnormal hypermethylation of SNRPN and ZAC1 resulted in 5.545 and 7.438 times higher risks of CHD with extracardiac malformations [144].

CITED2 (Cbp/p300 interacting transactivator with Glu/Asp rich carboxy-terminal domain 2) is a hypoxia-inducible transcriptional cofactor. CITED2 is critical for the development of the heart, and mice lacking CITED2 died in utero with various cardiac malformations, including atrial and ventricular septal defects, right-sided aortic arches, double-outlet right ventricle, common arterial trunk and overriding aorta [145–147]. An analysis of 392 patients with a broad range of CHDs found CITED2 mutations that did not exist in 192 control individuals. These mutations led to a significant loss in the HIF1A transcriptional repressive capacity of CITED2 and significantly diminished TFAP2C (a gene involved in early development, specifically morphogenesis) coactivation, providing evidence that CITED2 is a disease-causing gene for congenital heart malformations in humans, particularly septal defects and malrotations of the great arteries [148]. CITED2 gene promoter region methylation (decreased CITED2 transcriptional activity) as well as CITED2 gene mutations were discovered in another cohort of pediatric patients with CHD. Methylation in the promoter of CITED2 or loss-of-function mutations hindered the effect of HIF-1α overexpression. As a consequence, HIF-1α expression was enhanced, which could lead to disruption of apoptosis in the cardiac outflow tract, myocardial ischemia and hypoxia, inhibited migration and transition of neural crest cells, and abnormal heart formation as a result [149].

There is no doubt that whole genome DNA methylation profiling changes dynamically in the process of individual development and growth. Differential methylation profiling exists in different stages of cardiac development and CHD. Many differentially methylated genes or regions have been identified in CHD patients; however, most of them are inconsistent in different cases.

## Discussion

The heart is composed of several cell types, and the complexity of signal regulation in heart development and defects hinders the understanding, diagnosis and treatment of heart diseases. The diagnosis of CHD mainly depends on echocardiography of the fetus, and some defects in the heart require surgery. A better understanding of the etiology of CHD may allow diagnosis in earlier stages and even result in novel therapies. The DNA methylation profiling differences between the hearts of CHD patients and healthy persons offer a glimpse at the pathogenic mechanism of CHD, and advances in this field may provide new directions for drug development. In addition, differential DNA methylation in the placenta or blood in newborns and mothers may be used as a biomarker for diagnosis. Both the whole genome DNA methylation profile and the methylation level of some specific genes, such as ssTnI, are dynamic during cardiomyocyte development [100, 102]. It is possible to apply the dynamic DNA methylation as a marker of different stage of cardiomyocyte development and disease, or even monitor the efficacy of preventive strategies and treatment strategies. However, the dynamic nature of methylation in cardiomyocyte development has just begun to be understood. The application of differential DNA methylation as biomarker for diagnosis, drug target still demands tremendous efforts.

Due to the limitations of sequencing technologies in the past, many analyses of the differential methylation profile of the heart are based on the tissue level rather than the cell level. The DNA methylation profiles of cell types in the heart probably vary. Analysis at the tissue level may miss many differentially methylated genes due to the balance of differences by cell type. Single-cell DNA methylation sequencing technology provides a good solution. Future studies are needed to uncover the DNA methylomes of different cardiac cell types, for example, cardiomyocytes, fibroblasts, endothelial cells, and immune cells, to determine their epigenetic contribution to cardiac development and disease [150]. Another bottleneck in cardiac studies was the limitation of available heart tissue from healthy infants as a control for genome-wide analysis. Surrogate samples such as blood and placenta have been used to analyze the difference between CHD patients and healthy controls. It is helpful to identify DNA methylation biomarkers and serum molecules that could be used for risk estimation and detection of CHD.

Prenatal diagnosis of heart defects may improve clinical outcomes by adjusting medical management. It can be particularly important in the case of severe CHDs that may cause hypoxia and lead to organ damage or death in the absence of timely intervention [151–153]. Only 15% of mothers with a CHD-affected pregnancy reported receiving a prenatal diagnosis [153]. Efficient and sensitive detection technology can have major benefits. There are currently no biomarkers that can be used to detect heart defects. Multiple studies have identified differentially expressed circulating miRNAs as potential biomarkers for the diagnosis of CHD [154]. Differently methylated genes or regions among CHD patients and healthy persons or in various CHD types also provide potential biomarkers for diagnosis. However, the pathogenesis of CHD is highly complex, and differentially methylated genes vary in different CHD types, tissues, and even cases. It is still difficult to specify biomarkers as diagnostic criteria. Many studies with large populations, various types of CHD and samples from different tissues are still required for reliable identification and verification of biomarkers for diagnosis.

DNA demethylation drugs have been introduced as potential therapeutic agents for the treatment of human diseases [155]. Methylation intervention during pregnancy may reduce the incidence of CHD. In newborn CHD patients, these treatments are unlikely to restore heart structure through regulation of DNA methylation. However, DNA methylation is still a highly dynamic process during the postnatal growth of cardiomyocytes and their adaptation to pathological stress. For some of them, drugs regulating DNA methylation may decrease disease severity in adulthood. Current DNA demethylation drugs act on the overall methylation level. The development of drugs targeting DNA methylation of specific genes or regions has a long way to go.

## Conclusion

DNA methylation is an attractive topic in the study of complex diseases because it may be restored by environmental factors and dietary interventions. Major progress has been made in the understanding of DNA methylation involved in cardiac development and CHD pathogenesis. Several studies have identified dysregulated genome-wide DNA methylation profiles or differentially methylated genes. However, there is no clear evidence of which gene or combinations of genes with abnormal methylation can be used as diagnostic criteria or therapeutic targets for CHD. Maternal genome-wide DNA methylation patterns and DMRs deserve further exploration and discussion for early diagnosis and intervention. Continued investigation with new techniques makes the diagnosis and treatment of CHD by evaluating and modulating abnormal DNA methylation extremely promising.

## Abbreviations

ASD: Atrial septal defect
BAV: Bicuspid aortic valve
BSP: Bisulfite sequence PCR
DORV: Double outlet right ventricle
EM: Extracardiac malformations
HLHS: Hypoplastic left heart syndrome
LINE-1: Long interspersed nucleotide element-1
MSP: Methylation specific PCR
MTHFR: Methylene tetrahydrofolate reductase
MZ: Monozygotic
NGS: Next-generation sequencing
OFT: Outflow tract
PDA: Patent ductus arteriosus
PS: Pulmonary artery stenosis
PVS: Pulmonary valvular stenosis
RRBS: Reduced representation bisulfite sequencing
SHF: Second heart field TA: tricuspid atresia
TF: Transcription factor
TOF: Tetralogy of Fallot
TSS: Transcription start site
VSD: Ventricular septal defect
WGBS: Whole-genome bisulfite sequencing

## References

[1] Hoffman JI, Kaplan S (2002). The incidence of congenital heart disease. J Am Coll Cardiol.

[2] Vecoli C, Pulignani S, Foffa I, Andreassi MG (2014). Congenital heart disease: the crossroads of genetics, epigenetics and environment. Curr Genomics.

[3] Zhou P, Pu WT (2016). Recounting cardiac cellular composition. Circ Res.

[4] Hartman RJ, Rasmussen SA, Botto LD, Riehle-Colarusso T, Martin CL, Cragan JD, Shin M, Correa A (2011). The contribution of chromosomal abnormalities to congenital heart defects: a population-based study. Pediatr Cardiol.

[5] Lalani SR, Belmont JW (2014). Genetic basis of congenital cardiovascular malformations. Eur J Med Genet.

[6] Zaidi S, Choi M, Wakimoto H, Ma L, Jiang J, Overton JD, Romano-Adesman A, Bjornson RD, Breitbart RE, Brown KK, Carriero NJ, Cheung YH, Deanfield J, DePalma S, Fakhro KA, Glessner J, Hakonarson H, Italia MJ, Kaltman JR, Kaski J, Kim R, Kline JK, Lee T, Leipzig J, Lopez A, Mane SM, Mitchell LE, Newburger JW, Parfenov M, Pe'er I, Porter G, Roberts AE, Sachidanandam R, Sanders SJ, Seiden HS, State MW, Subramanian S, Tikhonova IR, Wang W, Warburton D, White PS, Williams IA, Zhao H, Seidman JG, Brueckner M, Chung WK, Gelb BD, Goldmuntz E, Seidman CE, Lifton RP (2013). De novo mutations in histone-modifying genes in congenital heart disease. Nature.

[7] Gupta RM (2013). Exome sequencing in congenital heart disease points to importance of DNA methylation. Circ Cardiovasc Genet.

[8] Jarrell DK, Lennon ML, Jacot JG (2019). Epigenetics and mechanobiology in heart development and congenital heart disease. Diseases.

[9] Weinhold B (2006). Epigenetics: the science of change. Environ Health Perspect.

[10] Hussain N (2012). Epigenetic influences that modulate infant growth, development, and disease. Antioxid Redox Signal.

[11] Moore-Morris T, van Vliet PP, Andelfinger G, Puceat M (2018). Role of epigenetics in cardiac development and congenital diseases. Physiol Rev.

[12] Bahado-Singh RO, Zaffra R, Albayarak S, Chelliah A, Bolinjkar R, Turkoglu O, Radhakrishna U (2016). Epigenetic markers for newborn congenital heart defect (CHD). J Matern Fetal Neonatal Med.

[13] Loscalzo J, Handy DE (2014). Epigenetic modifications: basic mechanisms and role in cardiovascular disease (2013 Grover Conference series). Pulm Circ.

[14] Szyf M (2011). The early life social environment and DNA methylation: DNA methylation mediating the long-term impact of social environments early in life. Epigenetics.

[15] Bird A (2002). DNA methylation patterns and epigenetic memory. Genes Dev.

[16] Shipony Z, Mukamel Z, Cohen NM, Landan G, Chomsky E, Zeliger SR, Fried YC, Ainbinder E, Friedman N, Tanay A (2014). Dynamic and static maintenance of epigenetic memory in pluripotent and somatic cells. Nature.

[17] Portela A, Esteller M (2010). Epigenetic modifications and human disease. Nat Biotechnol.

[18] Barchitta M, Maugeri A, Quattrocchi A, Barone G, Mazzoleni P, Catalfo A, De Guidi G, Iemmolo MG, Crimi N, Agodi A (2018). Mediterranean diet and particulate matter exposure are associated with LINE-1 methylation: results from a cross-sectional study in women. Front Genet.

[19] Barchitta M, Maugeri A, Magnano San Lio R, Favara G, La Rosa MC, La Mastra C, Quattrocchi A, Agodi A (2019). Dietary patterns are associated with leukocyte LINE-1 methylation in women: a cross-sectional study in Southern Italy. Nutrients.

[20] Maugeri A, Barchitta M, Magnano San Lio R, Favara G, La Rosa MC, La Mastra C, Basile G, Agodi A (2020). Adherence to the Mediterranean diet partially mediates socioeconomic differences in leukocyte LINE-1 methylation: evidence from a cross-sectional study in Italian women. Sci Rep.

[21] Maugeri A (2020). The effects of dietary interventions on DNA methylation: implications for obesity management. Int J Mol Sci.

[22] Maugeri A, Barchitta M (2020). How dietary factors affect DNA methylation: lesson from epidemiological studies. Medicina (Kaunas).

[23] Agodi A, Barchitta M, Quattrocchi A, Maugeri A, Canto C, Marchese AE, Vinciguerra M (2015). Low fruit consumption and folate deficiency are associated with LINE-1 hypomethylation in women of a cancer-free population. Genes Nutr.

[24] Yang M, Li W, Liu YY, Fu S, Qiu GB, Sun KL, Fu WN (2012). Promoter hypermethylation-induced transcriptional down-regulation of the gene MYCT1 in laryngeal squamous cell carcinoma. BMC Cancer.

[25] Jones PA (1999). The DNA methylation paradox. Trends Genet.

[26] Gouil Q, Keniry A (2019). Latest techniques to study DNA methylation. Essays Biochem.

[27] Peyser PA (1997). Genetic epidemiology of coronary artery disease. Epidemiol Rev.

[28] Moorman AF, Christoffels VM (2003). Cardiac chamber formation: development, genes, and evolution. Physiol Rev.

[29] Buckingham M, Meilhac S, Zaffran S (2005). Building the mammalian heart from two sources of myocardial cells. Nat Rev Genet.

[30] Xu H, Baldini A (2007). Genetic pathways to mammalian heart development: Recent progress from manipulation of the mouse genome. Semin Cell Dev Biol.

[31] Meilhac SM, Kelly RG, Rocancourt D, Eloy-Trinquet S, Nicolas JF, Buckingham ME (2003). A retrospective clonal analysis of the myocardium reveals two phases of clonal growth in the developing mouse heart. Development.

[32] Lin CJ, Lin CY, Chen CH, Zhou B, Chang CP (2012). Partitioning the heart: mechanisms of cardiac septation and valve development. Development.

[33] Sedmera D, Thompson RP (2011). Myocyte proliferation in the developing heart. Dev Dyn.

[34] Gunthel M, Barnett P, Christoffels VM (2018). Development, proliferation, and growth of the mammalian heart. Mol Ther.

[35] Desgrange A, Le Garrec JF, Meilhac SM (2018). Left-right asymmetry in heart development and disease: forming the right loop. Development.

[36] Foglia MJ, Poss KD (2016). Building and re-building the heart by cardiomyocyte proliferation. Development.

[37] Smits AM, Dronkers E, Goumans MJ (2018). The epicardium as a source of multipotent adult cardiac progenitor cells: their origin, role and fate. Pharmacol Res.

[38] Risebro CA, Riley PR (2006). Formation of the ventricles. Sci World J.

[39] Samsa LA, Yang B, Liu J (2013). Embryonic cardiac chamber maturation: Trabeculation, conduction, and cardiomyocyte proliferation. Am J Med Genet C Semin Med Genet.

[40] Anderson RH, Mori S, Spicer DE, Brown NA, Mohun TJ (2016). Development and Morphology of the Ventricular Outflow Tracts. World J Pediatr Congenit Heart Surg.

[41] Warnes CA, Liberthson R, Danielson GK, Dore A, Harris L, Hoffman JI, Somerville J, Williams RG, Webb GD (2001). Task force 1: the changing profile of congenital heart disease in adult life. J Am Coll Cardiol.

[42] Crucean A, Brawn WJ, Spicer DE, Franklin RC, Anderson RH (2015). Holes and channels between the ventricles revisited. Cardiol Young.

[43] Mai CT, Isenburg JL, Canfield MA, Meyer RE, Correa A, Alverson CJ, Lupo PJ, Riehle-Colarusso T, Cho SJ, Aggarwal D, Kirby RS, National Birth Defects Prevention N (2019). National population-based estimates for major birth defects, 2010–2014. Birth Defects Res.

[44] Kaplan S (1993). Congenital heart disease in adolescents and adults. Natural and postoperative history across age groups. Cardiol Clin.

[45] Van Praagh R (2009). The first Stella van Praagh memorial lecture: the history and anatomy of tetralogy of Fallot. Semin Thorac Cardiovasc Surg Pediatr Card Surg Annu.

[46] Bedard E, McCarthy KP, Dimopoulos K, Giannakoulas G, Gatzoulis MA, Ho SY (2009). Structural abnormalities of the pulmonary trunk in tetralogy of Fallot and potential clinical implications: a morphological study. J Am Coll Cardiol.

[47] Obler D, Juraszek AL, Smoot LB, Natowicz MR (2008). Double outlet right ventricle: aetiologies and associations. J Med Genet.

[48] McMahon CJ, Breathnach C, Betts DR, Sharkey FH, Greally MT (2015). De Novo interstitial deletion 13q33.3q34 in a male patient with double outlet right ventricle, microcephaly, dysmorphic craniofacial findings, and motor and developmental delay. Am J Med Genet A.

[49] Lyu G, Zhang C, Ling T, Liu R, Zong L, Guan Y, Huang X, Sun L, Zhang L, Li C, Nie Y, Tao W (2018). Genome and epigenome analysis of monozygotic twins discordant for congenital heart disease. BMC Genomics.

[50] Hartge DR, Niemeyer L, Axt-Fliedner R, Krapp M, Gembruch U, Germer U, Weichert J (2012). Prenatal detection and postnatal management of double outlet right ventricle (DORV) in 21 singleton pregnancies. J Matern Fetal Neonatal Med.

[51] Sheffield VC, Pierpont ME, Nishimura D, Beck JS, Burns TL, Berg MA, Stone EM, Patil SR, Lauer RM (1997). Identification of a complex congenital heart defect susceptibility locus by using DNA pooling and shared segment analysis. Hum Mol Genet.

[52] McGregor TL, Misri A, Bartlett J, Orabona G, Friedman RD, Sexton D, Maheshwari S, Morgan TM (2010). Consanguinity mapping of congenital heart disease in a South Indian population. PLoS ONE.

[53] Monteleone PL, Fagan LF (1969). Possible X-linked congenital heart disease. Circulation.

[54] Ferencz C, Neill CA, Boughman JA, Rubin JD, Brenner JI, Perry LW (1989). Congenital cardiovascular malformations associated with chromosome abnormalities: an epidemiologic study. J Pediatr.

[55] Trevisan P, Zen TD, Rosa RF, Silva JN, Koshiyama DB, Paskulin GA, Zen PR (2013). Chromosomal abnormalities in patients with congenital heart disease. Arq Bras Cardiol.

[56] Landis BJ, Cooper DS, Hinton RB (2016). CHD associated with syndromic diagnoses: peri-operative risk factors and early outcomes. Cardiol Young.

[57] Lin FJ, You LR, Yu CT, Hsu WH, Tsai MJ, Tsai SY (2012). Endocardial cushion morphogenesis and coronary vessel development require chicken ovalbumin upstream promoter-transcription factor II. Arterioscler Thromb Vasc Biol.

[58] Wu SP, Cheng CM, Lanz RB, Wang T, Respress JL, Ather S, Chen W, Tsai SJ, Wehrens XH, Tsai MJ, Tsai SY (2013). Atrial identity is determined by a COUP-TFII regulatory network. Dev Cell.

[59] Paige SL, Plonowska K, Xu A, Wu SM (2015). Molecular regulation of cardiomyocyte differentiation. Circ Res.

[60] Bruneau BG (2013). Signaling and transcriptional networks in heart development and regeneration. Cold Spring Harb Perspect Biol.

[61] Andersen TA, Troelsen Kde L, Larsen LA (2014). Of mice and men: molecular genetics of congenital heart disease. Cell Mol Life Sci.

[62] Sifrim A, Hitz MP, Wilsdon A, Breckpot J, Turki SH, Thienpont B, McRae J, Fitzgerald TW, Singh T, Swaminathan GJ, Prigmore E, Rajan D, Abdul-Khaliq H, Banka S, Bauer UM, Bentham J, Berger F, Bhattacharya S, BuLock F, Canham N, Colgiu IG, Cosgrove C, Cox H, Daehnert I, Daly A, Danesh J, Fryer A, Gewillig M, Hobson E, Hoff K, Homfray T, Study I, Kahlert AK, Ketley A, Kramer HH, Lachlan K, Lampe AK, Louw JJ, Manickara AK, Manase D, McCarthy KP, Metcalfe K, Moore C, Newbury-Ecob R, Omer SO, Ouwehand WH, Park SM, Parker MJ, Pickardt T, Pollard MO (2016). Distinct genetic architectures for syndromic and nonsyndromic congenital heart defects identified by exome sequencing. Nat Genet.

[63] Luxan G, D'Amato G, MacGrogan D, de la Pompa JL (2016). Endocardial notch signaling in cardiac development and disease. Circ Res.

[64] del Monte G, Casanova JC, Guadix JA, MacGrogan D, Burch JB, Perez-Pomares JM, de la Pompa JL (2011). Differential Notch signaling in the epicardium is required for cardiac inflow development and coronary vessel morphogenesis. Circ Res.

[65] Grieskamp T, Rudat C, Ludtke TH, Norden J, Kispert A (2011). Notch signaling regulates smooth muscle differentiation of epicardium-derived cells. Circ Res.

[66] Posch MG, Gramlich M, Sunde M, Schmitt KR, Lee SH, Richter S, Kersten A, Perrot A, Panek AN, Al Khatib IH, Nemer G, Megarbane A, Dietz R, Stiller B, Berger F, Harvey RP, Ozcelik C (2010). A gain-of-function TBX20 mutation causes congenital atrial septal defects, patent foramen ovale and cardiac valve defects. J Med Genet.

[67] Kirk EP, Sunde M, Costa MW, Rankin SA, Wolstein O, Castro ML, Butler TL, Hyun C, Guo G, Otway R, Mackay JP, Waddell LB, Cole AD, Hayward C, Keogh A, Macdonald P, Griffiths L, Fatkin D, Sholler GF, Zorn AM, Feneley MP, Winlaw DS, Harvey RP (2007). Mutations in cardiac T-box factor gene TBX20 are associated with diverse cardiac pathologies, including defects of septation and valvulogenesis and cardiomyopathy. Am J Hum Genet.

[68] Liu C, Shen A, Li X, Jiao W, Zhang X, Li Z (2008). T-box transcription factor TBX20 mutations in Chinese patients with congenital heart disease. Eur J Med Genet.

[69] Chen Y, Liu Q, Guo D (2020). Emerging coronaviruses: Genome structure, replication, and pathogenesis. J Med Virol.

[70] Dorr KM, Conlon FL (2019). Proteomic-based approaches to cardiac development and disease. Curr Opin Chem Biol.

[71] Patel SS, Burns TL (2013). Nongenetic risk factors and congenital heart defects. Pediatr Cardiol.

[72] Kaneko M, Kotake M, Bando H, Yamada T, Takemura H, Minamoto T (2016). Prognostic and predictive significance of long interspersed nucleotide element-1 methylation in advanced-stage colorectal cancer. BMC Cancer.

[73] Barchitta M, Quattrocchi A, Maugeri A, Canto C, La Rosa N, Cantarella MA, Spampinato G, Scalisi A, Agodi A (2017). LINE-1 hypermethylation in white blood cell DNA is associated with high-grade cervical intraepithelial neoplasia. BMC Cancer.

[74] Chowdhury S, Cleves MA, MacLeod SL, James SJ, Zhao W, Hobbs CA (2011). Maternal DNA hypomethylation and congenital heart defects. Birth Defects Res A Clin Mol Teratol.

[75] Bozovic IB, Stankovic A, Zivkovic M, Vranekovic J, Kapovic M, Brajenovic-Milic B (2015). Altered LINE-1 methylation in mothers of children with down syndrome. PLoS ONE.

[76] Babic Bozovic I, Stankovic A, Zivkovic M, Vranekovic J, Mahulja-Stamenkovic V, Brajenovic-Milic B (2019). Maternal LINE-1 DNA methylation and congenital heart defects in down syndrome. Front Genet.

[77] Chowdhury S, Erickson SW, MacLeod SL, Cleves MA, Hu P, Karim MA, Hobbs CA (2011). Maternal genome-wide DNA methylation patterns and congenital heart defects. PLoS ONE.

[78] Joziasse IC, van de Smagt JJ, Smith K, Bakkers J, Sieswerda GJ, Mulder BJ, Doevendans PA (2008). Genes in congenital heart disease: atrioventricular valve formation. Basic Res Cardiol.

[79] Garg V, Kathiriya IS, Barnes R, Schluterman MK, King IN, Butler CA, Rothrock CR, Eapen RS, Hirayama-Yamada K, Joo K, Matsuoka R, Cohen JC, Srivastava D (2003). GATA4 mutations cause human congenital heart defects and reveal an interaction with TBX5. Nature.

[80] Schleiffarth JR, Person AD, Martinsen BJ, Sukovich DJ, Neumann A, Baker CVH, Lohr JL, Cornfield DN, Ekker SC, Petryk A (2007). Wnt5a is required for cardiac outflow tract septation in mice. Pediatr Res.

[81] Joshi RO, Chellappan S, Kukshal P (2020). Exploring the Role of Maternal Nutritional Epigenetics in Congenital Heart Disease. Curr Dev Nutr.

[82] Friso S, Choi SW, Girelli D, Mason JB, Dolnikowski GG, Bagley PJ, Olivieri O, Jacques PF, Rosenberg IH, Corrocher R, Selhub J (2002). A common mutation in the 5,10-methylenetetrahydrofolate reductase gene affects genomic DNA methylation through an interaction with folate status. Proc Natl Acad Sci U S A.

[83] Hernandez-Robles J, Huhta JC, Leshko J (2005). Maternal MTHFR mutation and congenital heart defects. Am J Obstet Gynecol.

[84] Hobbs CA, Cleves MA, Karim MA, Zhao W, MacLeod SL (2010). Maternal folate-related gene environment interactions and congenital heart defects. Obstet Gynecol.

[85] Jaenisch R, Bird A (2003). Epigenetic regulation of gene expression: how the genome integrates intrinsic and environmental signals. Nat Genet.

[86] Doolin MT, Barbaux S, McDonnell M, Hoess K, Whitehead AS, Mitchell LE (2002). Maternal genetic effects, exerted by genes involved in homocysteine remethylation, influence the risk of spina bifida. Am J Hum Genet.

[87] Hajkova P, Erhardt S, Lane N, Haaf T, El-Maarri O, Reik W, Walter J, Surani MA (2002). Epigenetic reprogramming in mouse primordial germ cells. Mech Dev.

[88] Lees-Murdock DJ, De Felici M, Walsh CP (2003). Methylation dynamics of repetitive DNA elements in the mouse germ cell lineage. Genomics.

[89] Sasaki H, Matsui Y (2008). Epigenetic events in mammalian germ-cell development: reprogramming and beyond. Nat Rev Genet.

[90] Mayer W, Niveleau A, Walter J, Fundele R, Haaf T. Demethylation of the zygotic paternal genome. Nature. 2000;403(6769):501–2. 10.1038/35000656 10676950

[91] Oswald J, Engemann S, Lane N, Mayer W, Olek A, Fundele R, Dean W, Reik W, Walter J. Active demethylation of the paternal genome in the mouse zygote. Curr Biol. 2000;10(8):475–8. 10.1016/s0960-9822(00)00448-6 10801417

[92] Santos F, Hendrich B, Reik W, Dean W (2002). Dynamic reprogramming of DNA methylation in the early mouse embryo. Dev Biol.

[93] Fulka H, Mrazek M, Tepla O, Fulka J (2004). DNA methylation pattern in human zygotes and developing embryos. Reproduction.

[94] Zhu P, Guo H, Ren Y, Hou Y, Dong J, Li R, Lian Y, Fan X, Hu B, Gao Y, Wang X, Wei Y, Liu P, Yan J, Ren X, Yuan P, Yuan Y, Yan Z, Wen L, Yan L, Qiao J, Tang F (2018). Single-cell DNA methylome sequencing of human preimplantation embryos. Nat Genet.

[95] Wang L, Zhang J, Duan J, Gao X, Zhu W, Lu X, Yang L, Zhang J, Li G, Ci W, Li W, Zhou Q, Aluru N, Tang F, He C, Huang X, Liu J (2014). Programming and inheritance of parental DNA methylomes in mammals. Cell.

[96] Seisenberger S, Andrews S, Krueger F, Arand J, Walter J, Santos F, Popp C, Thienpont B, Dean W, Reik W (2012). The dynamics of genome-wide DNA methylation reprogramming in mouse primordial germ cells. Mol Cell.

[97] Hon GC, Rajagopal N, Shen Y, McCleary DF, Yue F, Dang MD, Ren B (2013). Epigenetic memory at embryonic enhancers identified in DNA methylation maps from adult mouse tissues. Nat Genet.

[98] Varley KE, Gertz J, Bowling KM, Parker SL, Reddy TE, Pauli-Behn F, Cross MK, Williams BA, Stamatoyannopoulos JA, Crawford GE, Absher DM, Wold BJ, Myers RM (2013). Dynamic DNA methylation across diverse human cell lines and tissues. Genome Res.

[99] Schultz MD, He Y, Whitaker JW, Hariharan M, Mukamel EA, Leung D, Rajagopal N, Nery JR, Urich MA, Chen H, Lin S, Lin Y, Jung I, Schmitt AD, Selvaraj S, Ren B, Sejnowski TJ, Wang W, Ecker JR (2015). Human body epigenome maps reveal noncanonical DNA methylation variation. Nature.

[100] Chamberlain AA, Lin M, Lister RL, Maslov AA, Wang Y, Suzuki M, Wu B, Greally JM, Zheng D (2014). DNA methylation is developmentally regulated for genes essential for cardiogenesis. JAHA.

[101] Gilsbach R, Preissl S, Gruning BA, Schnick T, Burger L, Benes V, Wurch A, Bonisch U, Gunther S, Backofen R, Fleischmann BK, Schubeler D, Hein L (2014). Dynamic DNA methylation orchestrates cardiomyocyte development, maturation and disease. Nat Commun.

[102] Xu Y, Liu L, Pan B, Zhu J, Nan C, Huang X, Tian J (2015). DNA methylation regulates mouse cardiac myofibril gene expression during heart development. J Biomed Sci.

[103] Sim CB, Ziemann M, Kaspi A, Harikrishnan KN, Ooi J, Khurana I, Chang L, Hudson JE, El-Osta A, Porrello ER (2015). Dynamic changes in the cardiac methylome during postnatal development. FASEB J.

[104] Lan Y, Evans T (2019). Epigenetic regulation of cardiac development and disease through DNA methylation. J Life Sci (Westlake Village).

[105] Michels KB, Binder AM, Dedeurwaerder S, Epstein CB, Greally JM, Gut I, Houseman EA, Izzi B, Kelsey KT, Meissner A, Milosavljevic A, Siegmund KD, Bock C, Irizarry RA (2013). Recommendations for the design and analysis of epigenome-wide association studies. Nat Methods.

[106] Bergmann O, Zdunek S, Felker A, Salehpour M, Alkass K, Bernard S, Sjostrom SL, Szewczykowska M, Jackowska T, Dos Remedios C, Malm T, Andra M, Jashari R, Nyengaard JR, Possnert G, Jovinge S, Druid H, Frisen J (2015). Dynamics of cell generation and turnover in the human heart. Cell.

[107] Gilsbach R, Schwaderer M, Preissl S, Gruning BA, Kranzhofer D, Schneider P, Nuhrenberg TG, Mulero-Navarro S, Weichenhan D, Braun C, Dressen M, Jacobs AR, Lahm H, Doenst T, Backofen R, Krane M, Gelb BD, Hein L (2018). Distinct epigenetic programs regulate cardiac myocyte development and disease in the human heart in vivo. Nat Commun.

[108] Sheng W, Qian Y, Wang H, Ma X, Zhang P, Chen L, Ma D, Huang G (2013). Association between mRNA levels of DNMT1, DNMT3A, DNMT3B, MBD2 and LINE-1 methylation status in infants with tetralogy of Fallot. Int J Mol Med.

[109] Grunert M, Dorn C, Cui H, Dunkel I, Schulz K, Schoenhals S, Sun W, Berger F, Chen W, Sperling SR (2016). Comparative DNA methylation and gene expression analysis identifies novel genes for structural congenital heart diseases. Cardiovasc Res.

[110] Johnson SE, Rothstein JL, Knowles BB (1994). Expression of epidermal growth factor family gene members in early mouse development. Dev Dyn.

[111] Behrens AN, Ren Y, Ferdous A, Garry DJ, Martin CM (2012). Nkx2-5 regulates Tdgf1 (Cripto) early during cardiac development. J Clin Exp Cardiolog Suppl.

[112] Serra-Juhe C, Cusco I, Homs A, Flores R, Toran N, Perez-Jurado LA (2015). DNA methylation abnormalities in congenital heart disease. Epigenetics.

[113] Stankunas K, Hang CT, Tsun ZY, Chen H, Lee NV, Wu JI, Shang C, Bayle JH, Shou W, Iruela-Arispe ML, Chang CP (2008). Endocardial Brg1 represses ADAMTS1 to maintain the microenvironment for myocardial morphogenesis. Dev Cell.

[114] Bultman S, Gebuhr T, Yee D, La Mantia C, Nicholson J, Gilliam A, Randazzo F, Metzger D, Chambon P, Crabtree G, Magnuson T (2000). A Brg1 null mutation in the mouse reveals functional differences among mammalian SWI/SNF complexes. Mol Cell.

[115] Kosho T, Okamoto N, Ohashi H, Tsurusaki Y, Imai Y, Hibi-Ko Y, Kawame H, Homma T, Tanabe S, Kato M, Hiraki Y, Yamagata T, Yano S, Sakazume S, Ishii T, Nagai T, Ohta T, Niikawa N, Mizuno S, Kaname T, Naritomi K, Narumi Y, Wakui K, Fukushima Y, Miyatake S, Mizuguchi T, Saitsu H, Miyake N, Matsumoto N (2013). Clinical correlations of mutations affecting six components of the SWI/SNF complex: detailed description of 21 patients and a review of the literature. Am J Med Genet A.

[116] Qian Y, Xiao D, Guo X, Chen H, Hao L, Ma X, Huang G, Ma D, Wang H (2017). Hypomethylation and decreased expression of BRG1 in the myocardium of patients with congenital heart disease. Birth Defects Res.

[117] Mehta G, Kumarasamy S, Wu J, Walsh A, Liu L, Williams K, Joe B, de la Serna IL (2015). MITF interacts with the SWI/SNF subunit, BRG1, to promote GATA4 expression in cardiac hypertrophy. J Mol Cell Cardiol.

[118] Dobosz A, Grabowska A, Bik-Multanowski M (2019). Hypermethylation of NRG1 gene correlates with the presence of heart defects in Down's syndrome. J Genet.

[119] Sheng W, Wang H, Ma X, Qian Y, Zhang P, Wu Y, Zheng F, Chen L, Huang G, Ma D (2012). LINE-1 methylation status and its association with tetralogy of fallot in infants. BMC Med Genomics.

[120] Aporntewan C, Phokaew C, Piriyapongsa J, Ngamphiw C, Ittiwut C, Tongsima S, Mutirangura A (2011). Hypomethylation of intragenic LINE-1 represses transcription in cancer cells through AGO2. PLoS ONE.

[121] Sheng W, Qian Y, Wang H, Ma X, Zhang P, Diao L, An Q, Chen L, Ma D, Huang G (2013). DNA methylation status of NKX2-5, GATA4 and HAND1 in patients with tetralogy of fallot. BMC Med Genomics.

[122] Bruneau BG (2008). The developmental genetics of congenital heart disease. Nature.

[123] Sheng W, Qian Y, Zhang P, Wu Y, Wang H, Ma X, Chen L, Ma D, Huang G (2014). Association of promoter methylation statuses of congenital heart defect candidate genes with Tetralogy of Fallot. J Transl Med.

[124] Radhakrishna U, Albayrak S, Alpay-Savasan Z, Zeb A, Turkoglu O, Sobolewski P, Bahado-Singh RO (2016). Genome-wide DNA methylation analysis and epigenetic variations associated with congenital aortic valve stenosis (AVS). PLoS ONE.

[125] Radhakrishna U, Vishweswaraiah S, Veerappa AM, Zafra R, Albayrak S, Sitharam PH, Saiyed NM, Mishra NK, Guda C, Bahado-Singh R (2018). Newborn blood DNA epigenetic variations and signaling pathway genes associated with Tetralogy of Fallot (TOF). PLoS ONE.

[126] Arrington CB, Bleyl SB, Matsunami N, Bonnell GD, Otterud BE, Nielsen DC, Stevens J, Levy S, Leppert MF, Bowles NE (2012). Exome analysis of a family with pleiotropic congenital heart disease. Circ Cardiovasc Genet.

[127] Click ES, Cox B, Olson SB, Grompe M, Akkari Y, Moreau LA, Shimamura A, Sternen DL, Liu YJ, Leppig KA, Matthews DC, Parisi MA (2011). Fanconi anemia-like presentation in an infant with constitutional deletion of 21q including the RUNX1 gene. Am J Med Genet A.

[128] Conrad M, Jakupoglu C, Moreno SG, Lippl S, Banjac A, Schneider M, Beck H, Hatzopoulos AK, Just U, Sinowatz F, Schmahl W, Chien KR, Wurst W, Bornkamm GW, Brielmeier M (2004). Essential role for mitochondrial thioredoxin reductase in hematopoiesis, heart development, and heart function. Mol Cell Biol.

[129] Liu HK, Wang CT, Zhang SZ, Xiao CY, Li XF, Zhang KL, Zhang L, Su ZG, Ma YX, Zhou B, Zheng KQ, Li GX (2004). Association of APOA5 gene single nucleotide polymorphism with levels of lipids and coronary heart disease in Chinese. Zhonghua Yi Xue Yi Chuan Xue Za Zhi.

[130] Huang CC, Fornage M, Lloyd-Jones DM, Wei GS, Boerwinkle E, Liu K (2009). Longitudinal association of PCSK9 sequence variations with low-density lipoprotein cholesterol levels: the Coronary Artery Risk Development in Young Adults Study. Circ Cardiovasc Genet.

[131] Cohen JC, Boerwinkle E, Mosley TH, Hobbs HH (2006). Sequence variations in PCSK9, low LDL, and protection against coronary heart disease. N Engl J Med.

[132] Bahado-Singh RO, Vishweswaraiah S, Aydas B, Yilmaz A, Saiyed NM, Mishra NK, Guda C, Radhakrishna U (2020). Precision cardiovascular medicine: artificial intelligence and epigenetics for the pathogenesis and prediction of coarctation in neonates. J Matern Fetal Neonatal Med.

[133] Laufer BI, Hwang H, Jianu JM, Mordaunt CE, Korf IF, Hertz-Picciotto I, LaSalle JM (2021). Low-pass whole genome bisulfite sequencing of neonatal dried blood spots identifies a role for RUNX1 in Down syndrome DNA methylation profiles. Hum Mol Genet.

[134] Yuan W, Xia Y, Bell CG, Yet I, Ferreira T, Ward KJ, Gao F, Loomis AK, Hyde CL, Wu H, Lu H, Liu Y, Small KS, Vinuela A, Morris AP, Berdasco M, Esteller M, Brosnan MJ, Deloukas P, McCarthy MI, John SL, Bell JT, Wang J, Spector TD (2014). An integrated epigenomic analysis for type 2 diabetes susceptibility loci in monozygotic twins. Nat Commun.

[135] Wong CC, Meaburn EL, Ronald A, Price TS, Jeffries AR, Schalkwyk LC, Plomin R, Mill J (2014). Methylomic analysis of monozygotic twins discordant for autism spectrum disorder and related behavioural traits. Mol Psychiatry.

[136] Castillo-Fernandez JE, Spector TD, Bell JT (2014). Epigenetics of discordant monozygotic twins: implications for disease. Genome Med.

[137] Fraga MF, Ballestar E, Paz MF, Ropero S, Setien F, Ballestar ML, Heine-Suner D, Cigudosa JC, Urioste M, Benitez J, Boix-Chornet M, Sanchez-Aguilera A, Ling C, Carlsson E, Poulsen P, Vaag A, Stephan Z, Spector TD, Wu YZ, Plass C, Esteller M (2005). Epigenetic differences arise during the lifetime of monozygotic twins. Proc Natl Acad Sci U S A.

[138] Al Turki S, Manickaraj AK, Mercer CL, Gerety SS, Hitz MP, Lindsay S, D’Alessandro LC, Swaminathan GJ, Bentham J, Arndt AK, Louw J, Low J, Breckpot J, Gewillig M, Thienpont B, Abdul-Khaliq H, Harnack C, Hoff K, Kramer HH, Schubert S, Siebert R, Toka O, Cosgrove C, Watkins H, Lucassen AM, O'Kelly IM, Salmon AP, Bu'lock FA, Granados-Riveron J, Setchfield K, Thornborough C, Brook JD, Mulder B, Klaassen S, Bhattacharya S, Devriendt K, Fitzpatrick DF, Consortium UK, Wilson DI, Mital S, Hurles ME. Rare variants in NR2F2 cause congenital heart defects in humans. Am J Hum Genet. 2014;94(4):574-85. 10.1016/j.ajhg.2014.03.007 PMC3980509 24702954

[139] Grunert M, Appelt S, Grossfeld P, Sperling SR (2020). The needle in the haystack-searching for genetic and epigenetic differences in monozygotic twins discordant for tetralogy of fallot. J Cardiovasc Dev Dis.

[140] Sailani MR, Santoni FA, Letourneau A, Borel C, Makrythanasis P, Hibaoui Y, Popadin K, Bonilla X, Guipponi M, Gehrig C, Vannier A, Carre-Pigeon F, Feki A, Nizetic D, Antonarakis SE (2015). DNA-methylation patterns in trisomy 21 using cells from monozygotic twins. Plos One.

[141] Radhakrishna U, Albayrak S, Zafra R, Baraa A, Vishweswaraiah S, Veerappa AM, Mahishi D, Saiyed N, Mishra NK, Guda C, Ali-Fehmi R, Bahado-Singh RO (2019). Placental epigenetics for evaluation of fetal congenital heart defects: Ventricular Septal Defect (VSD). PLoS ONE.

[142] Bahado-Singh R, Vishweswaraiah S, Mishra NK, Guda C, Radhakrishna U (2020). Placental DNA methylation changes in detection of tetralogy of Fallot. Ultrasound Obstet Gynecol.

[143] Chang S, Wang Y, Xin Y, Wang S, Luo Y, Wang L, Zhang H, Li J (2021). DNA methylation abnormalities of imprinted genes in congenital heart disease: a pilot study. BMC Med Genomics.

[144] Zhao X, Chang S, Liu X, Wang S, Zhang Y, Lu X, Zhang T, Zhang H, Wang L (2020). Imprinting aberrations of SNRPN, ZAC1 and INPP5F genes involved in the pathogenesis of congenital heart disease with extracardiac malformations. J Cell Mol Med.

[145] Bamforth SD, Braganca J, Eloranta JJ, Murdoch JN, Marques FI, Kranc KR, Farza H, Henderson DJ, Hurst HC, Bhattacharya S (2001). Cardiac malformations, adrenal agenesis, neural crest defects and exencephaly in mice lacking Cited2, a new Tfap2 co-activator. Nat Genet.

[146] Weninger WJ, Floro KL, Bennett MB, Withington SL, Preis JI, Barbera JPM, Mohun TJ, Dunwoodie SL (2005). Cited2 is required both for heart morphogenesis and establishment of the left-right axis in mouse development. Development.

[147] Bamforth SD, Braganca J, Farthing CR, Schneider JE, Broadbent C, Michell AC, Clarke K, Neubauer S, Norris D, Brown NA, Anderson RH, Bhattacharya S (2004). Cited2 controls left-right patterning and heart development through a Nodal-Pitx2c pathway. Nat Genet.

[148] Sperling S, Grimm CH, Dunkel I, Mebus S, Sperling HP, Ebner A, Galli R, Lehrach H, Fusch C, Berger F, Hammer S (2005). Identification and functional analysis of CITED2 mutations in patients with congenital heart defects. Hum Mutat.

[149] Xu M, Wu X, Li Y, Yang X, Hu J, Zheng M, Tian J (2014). CITED2 mutation and methylation in children with congenital heart disease. J Biomed Sci.

[150] Cui Y, Zheng Y, Liu X, Yan L, Fan X, Yong J, Hu Y, Dong J, Li Q, Wu X, Gao S, Li J, Wen L, Qiao J, Tang F (2019). Single-Cell Transcriptome Analysis Maps the Developmental Track of the Human Heart. Cell Rep.

[151] Wren C, Reinhardt Z, Khawaja K (2008). Twenty-year trends in diagnosis of life-threatening neonatal cardiovascular malformations. Arch Dis Child Fetal Neonatal Ed.

[152] Mahle WT, Newburger JW, Matherne GP, Smith FC, Hoke TR, Koppel R, Gidding SS, Beekman RH, 3rd, Grosse SD, American Heart Association Congenital Heart Defects Committee of the Council on Cardiovascular Disease in the Young CoCN, Interdisciplinary Council on Quality of C, Outcomes R, American Academy of Pediatrics Section on C, Cardiac S, Committee on F, Newborn. Role of pulse oximetry in examining newborns for congenital heart disease: a scientific statement from the American Heart Association and American Academy of Pediatrics. Circulation. 2009;120:447-58 10.1161/CIRCULATIONAHA.109.192576 19581492

[153] Ailes EC, Gilboa SM, Riehle-Colarusso T, Johnson CY, Hobbs CA, Correa A, Honein MA, Prevention NBD (2014). Prenatal diagnosis of nonsyndromic congenital heart defects. Prenat Diagn.

[154] Xie WQ, Zhou L, Chen Y, Ni B (2016). Circulating microRNAs as potential biomarkers for diagnosis of congenital heart defects. World J Emerg Med.

[155] Yang X, Lay F, Han H, Jones PA (2010). Targeting DNA methylation for epigenetic therapy. Trends Pharmacol Sci.

[156] Sailani MR, Santoni FA, Letourneau A, Borel C, Makrythanasis P, Hibaoui Y, Popadin K, Bonilla X, Guipponi M, Gehrig C, Vannier A, Carre-Pigeon F, Feki A, Nizetic D, Antonarakis SE (2015). DNA-Methylation Patterns in Trisomy 21 Using Cells from Monozygotic Twins. PLoS ONE.

